# Mapping the Neurophysiological Link Between Voice and Autonomic Function: A Scoping Review

**DOI:** 10.3390/biology14101382

**Published:** 2025-10-10

**Authors:** Carmen Morales-Luque, Laura Carrillo-Franco, Manuel Víctor López-González, Marta González-García, Marc Stefan Dawid-Milner

**Affiliations:** 1 Department of Human Physiology, Faculty of Medicine, University of Málaga, 29010 Málaga, Spain; laura_carrillo@uma.es (L.C.-F.); mgonzalezgarcia@uma.es (M.G.-G.); msdawid@uma.es (M.S.D.-M.); 2 Biomedical Research Institute of Málaga (IBIMA Plataforma BIONAND), 29010 Málaga, Spain; 3 Unit of Neurophysiology of the Autonomic Nervous System (CIMES), University of Málaga, 29010 Málaga, Spain

**Keywords:** voice production, voice use, vocal tasks, singing, vocal load, functional voice disorders, autonomic nervous system, heart rate variability, electrodermal activity, stress response

## Abstract

**Simple Summary:**

Speaking and singing places complex demands on the body, requiring coordination between breathing, muscular control, and the autonomic nervous system (ANS), which regulates functions such as heart rate (HR) and blood pressure (BP) and mediates stress responses. In individuals with high vocal demands—such as singers, teachers, or actors—this interaction may influence autonomic balance, even in the absence of manifest voice disorders. This review examines how voice production and autonomic regulation are interconnected, focusing on scientific studies that assessed both processes simultaneously.

**Abstract:**

Vocal production requires the coordinated control of respiratory, laryngeal, and autonomic systems. In individuals with high vocal demand, this physiological load may influence autonomic regulation, even in the absence of voice disorders. This scoping review systematically mapped current evidence on the relationship between voice production and autonomic nervous system (ANS) activity in adults, focusing exclusively on studies that assessed both systems simultaneously. A systematic search was conducted in PubMed, Scopus, Web of Science, Embase, and CINAHL, following PRISMA-ScR guidelines. Eligible studies included adults performing structured vocal tasks with concurrent autonomic measurements. Data were extracted and synthesized descriptively. Fifteen studies met the inclusion criteria. Most involved healthy adults with high vocal demand, while some included participants with subclinical or functional voice traits. Vocal tasks ranged from singing and sustained phonation to speech under cognitive or emotional load. Autonomic measures included heart rate (HR), heart rate variability (HRV), and electrodermal activity (EDA), among others. Four thematic trends emerged: autonomic synchronization during group vocalization; modulation of autonomic tone by vocal rhythm and structure; voice–ANS interplay under stress; and physiological coupling in hyperfunctional vocal behaviours. This review’s findings suggest that vocal activity can modulate autonomic function, supporting the potential integration of autonomic markers into experimental and clinical voice research.

## 1. Introduction

Vocal production requires precise coordination of the respiratory, laryngeal, and supralaryngeal systems, regulated by voluntary neuromuscular control and modulated by the autonomic nervous system (ANS). This function, essential for both spoken and sung communication, depends on fine motor control, aerodynamic balance, and continuous biomechanical adjustments to maintain efficient phonation [1,2,3].

These physiological demands increase substantially in individuals who rely heavily on their voice for professional or intensive use. In such high-vocal-load contexts, functional overload may occur, even in the absence of structural pathology, potentially leading to reduced vocal endurance, phonatory instability, or changes in voice quality. Several studies have documented this phenomenon across different populations. For example, singing students exhibit a high prevalence of laryngeal discomfort and potentially detrimental vocal behaviors [4]. Endoscopic assessments in professional singers without self-reported symptoms have revealed unexpectedly high rates of laryngeal abnormalities, and similar findings have been reported in pre-professional musical theatre students, suggesting subclinical vocal strain or chronic biomechanical stress [5,6]. Wind instrument players also face comparable demands, as the generation and regulation of subglottal pressure during performances requires active laryngeal control and precise respiratory–laryngeal coordination, placing stresses on the vocal tract similar to those experienced by singers and other professional voice users [7].

The physiological strain experienced by individuals with intensive vocal usage underscores the importance of involuntary regulatory systems in sustaining vocal function. Although phonation is initiated through voluntary motor control, its efficiency depends on automatic processes governed by the ANS [8]. The ANS continuously modulates parameters such as heart rate (HR), blood pressure (BP), pulmonary ventilation, and subglottal pressure, which together provide the physiological support required for effective voice production. For example, stable subglottal pressure depends on cardiorespiratory coupling and baroreceptor reflexes, while autonomic adjustments in HR and BP maintain adequate perfusion during vocal effort [9,10]. ANS enables rapid adjustments, including changes in HR, vascularity tone and ventilatory drive, which preserve homeostasis while adapting to changing behavioral challenges [11,12,13].

In this context, autonomic regulation has most commonly been evaluated through derived physiological indices that capture distinct dimensions of parasympathetic and sympathetic activity. Respiratory sinus arrhythmia (RSA), calculated from the covariation between respiration and cardiac period, reflects parasympathetic modulation and has been associated with emotion regulation [14]. Heart rate variability (HRV), obtained from electrocardiogram (ECG)-derived interbeat intervals, provides additional insight: its high-frequency component (HF) indexes parasympathetic influences, while the low-frequency component (LF) reflects both parasympathetic and sympathetic contributions. The LF/HF ratio has often been used to represent sympathovagal balance, although its interpretation remains debated [15]. By contrast, electrodermal activity (EDA), most commonly quantified through skin conductance responses (SCR), is directly measured and constitutes a sensitive marker of sympathetic activation, widely applied in psychophysiological research [16]. Taken together, RSA, HRV, and EDA represent some of the most frequently employed autonomic indices, among others, and are generally interpreted as complementary indicators of autonomic function, providing a framework to investigate how central autonomic networks contribute to complex motor behaviors such as vocal production.

On this basis, empirical studies have provided direct evidence that cardiovascular dynamics and autonomic regulation can modulate vocal function. Early findings indicated that the cardiac cycle exerts a modest but consistent influence on fundamental frequency (F_0_) [17]. Along these lines, sympathetic activation could induce changes in intrinsic laryngeal muscle activity during a silent speech-preparation task, without active phonation or functional voice assessment [18]. Distinct autonomic responses have further been observed in singing students during live performances, depending on cognitive performance levels. In addition, functional vocal complaints in teachers have been associated with reduced HRV [19,20].

Despite these meaningful associations, research in this field lacks conceptual clarity and exhibits notable methodological heterogeneity. Some studies have examined acoustic and autonomic parameters in isolation, relied on self-reported autonomic symptoms, or inferred autonomic involvement indirectly from task design [21,22,23]. Overall, this remains a fragmented area of study, making it difficult to identify consistent patterns across isolated investigations.

To advance this line of research, the present scoping review aims to map the available scientific evidence on the interaction between vocal production and autonomic regulation in adults, focusing exclusively on studies that evaluate both systems simultaneously and in an integrated manner. Emphasis is placed on research that records primary physiological signals and derives standardized autonomic indices in parallel with direct voice assessment, thereby providing a more comprehensive view of the phenomenon. The objective is to broaden the scope of investigation and examine how the ANS interacts with, and exerts regulatory influence on, different forms of vocalization—whether spoken or sung—in order to identify the key patterns of this interplay. Ultimately, the review seeks to provide a clear and systematic synthesis of the existing evidence, facilitating the identification of consistent findings and supporting the development of explanatory models that frame the voice as a motor behaviour modulated by the ANS.

## 2. Materials and Methods

### 2.1. Methodological Framework and Objectives

This scoping review was conducted in accordance with the PRISMA Extension for Scoping Reviews (PRISMA-ScR) guidelines and following the methodological recommendations of the Joanna Briggs Institute (JBI), which provide a standardized framework for mapping the breadth and nature of evidence across a defined field of study. The review protocol was prospectively registered on the Open Science Framework (OSF) and is publicly available at https://doi.org/10.17605/OSF.IO/AXS8F (accessed on 7 May 2025).

The review systematically mapped the scientific literature on the modulation of the ANS during spoken and sung voice production in adult populations. Focus was placed on adult populations engaged in structured vocal activity—primarily healthy individuals with high vocal demands, such as opera singers, amateur and professional choristers, music students, and performing artists. While some studies included individuals with subclinical or self-reported voice difficulties, the majority focused on non-pathological voice users, enabling the exploration of autonomic modulation under various vocal demands and ecological or experimental conditions.

Specifically, this scoping review aimed to: (1) identify the autonomic and cardiovascular parameters most frequently derived from primary physiological recordings during voice-related tasks (e.g., HRV, EDA, blood pressure); (2) examine the methodological approaches employed to investigate voice–ANS interactions in both experimental and clinical contexts, with particular attention to studies that assessed both systems simultaneously and in an integrated manner; and (3) highlight key gaps in current knowledge and outline future directions for research at the intersection of voice science, physiology, and autonomic regulation.

### 2.2. Inclusion Criteria and Conceptual Scope

Eligibility criteria were defined using the Population–Concept–Context (PCC) framework and were further refined during full-text screening based on specific methodological thresholds.

**Population (P)**

- Studies were eligible if they included adults (≥18 years) who met at least one of the following criteria:Demonstrated high vocal demand, such as singers, actors, choir members, teachers, or performing arts students, provided they actively engaged in structured vocal tasks within experimental or ecological settings.
- Presented with functional dysphonia (either clinically diagnosed or subclinical), when accompanied by monitored vocal activity and relevant physiological measurements.

Studies were excluded if participants met any of the following conditions, which were considered incompatible with the scope of the review:

- Individuals with chronic cardiovascular conditions (e.g., heart failure), due to the risk of confounding autonomic responses unrelated to vocal activity.
- Studies in which participants did not perform vocalizations, or where voice was used solely as a stimulus (e.g., to elicit stress) without acoustic or physiological analysis.

**Concept (C)**

Eligible studies assessed autonomic or cardiovascular responses during voice production. Accepted measures included:

- Direct and derived autonomic indices: such as HR, HRV, RSA, EDA, BPV, baroreflex sensitivity.
- Indirect markers: such as salivary cortisol, when clearly associated with autonomic regulation and when voice production was a central element of the protocol.

Excluded studies that reported only general physiological data alone (e.g., HR, respiratory rate) without autonomic interpretation or without a functional analysis of vocal performance.

**Context (C)**

Contexts were eligible when they involved structured or naturalistic vocal tasks allowing simultaneous assessment of autonomic variables:

- Ecological settings such as live performances, oral examinations, or rehearsals.
- Controlled experimental protocols involving structured vocal tasks, provided that both vocal behaviour and autonomic activity were objectively measured and jointly interpreted.

Studies were excluded if vocalization was unmonitored or used merely as a generic physical task, without any direct interpretation of voice–ANS interaction.

Only peer-reviewed journal articles published between January 2010 and April 2025 were considered, as the consistent use of objective autonomic measures in voice research (e.g., HRV, EDA, subglottic pressure) became more widespread and methodologically standardized during the past decade. Eligible studies had to be written in English. These limits were established to ensure methodological quality, accessibility of content, and relevance to current scientific standards in voice research and autonomic physiology.

In all cases, studies were required to report objective physiological recordings. In studies assessing multiple outcomes, only those explicitly linking vocal activity with ANS responses were extracted and analysed. Vocal tasks embedded within broader experimental paradigms—such as those involving cognitive load, emotional processing, or stress—were included only when the voice–ANS relationship was clearly integrated into the study’s analytical framework.

### 2.3. Search Strategy and Study Selection Process

The literature search was conducted across five major biomedical databases: PubMed, Scopus, Web of Science, Embase, and CINAHL. The search strategy was developed in alignment with the predefined inclusion criteria and conceptually structured around the PCC model. Key terms were derived from each component and combined using Boolean operators, then adapted to the syntax and filtering options specific to each database. A list of representative keywords organized according to the PCC framework is provided in Table 1.

Initial screening of titles and abstracts was carried out directly within the database interfaces. Records deemed potentially relevant were exported to Mendeley Reference Manager, where duplicates were identified and removed. The remaining unique records were organized in Excel spreadsheets for full-text eligibility assessment. The search process was conducted between March and May 2025. As a quality control measure, reference lists of included studies were manually reviewed to verify completeness, although no additional eligible articles were retrieved through this process.

Study selection was performed independently by two reviewers following a structured two-phase process: (1) screening of titles and abstracts and (2) full-text review based on predefined eligibility criteria. Discrepancies were resolved through discussion, and a third reviewer was consulted when necessary.

### 2.4. Data Extraction and Variables Collected

Once the final set of studies was confirmed, relevant data were extracted by one reviewer using a predefined format tailored to the aims of this review. The structure and categories of this charting tool were agreed upon by the research team. The extracted information was subsequently cross-checked by a second reviewer to ensure accuracy and consistency. No automated extraction tools were used, and no contact with original authors was required. The following variables were collected from each included study:

- **Bibliographic information**: authors and year of publication.
- **Study design**: methodological classification (e.g., experimental, cross-sectional, observational, longitudinal).
- **Study population**: number of participants, demographic characteristics (e.g., age, sex), and vocal profile (e.g., singers, teachers, individuals with functional voice disorders).
- **Study objective**: main aim or hypothesis related to the relationship between vocal production and autonomic regulation.

**Autonomic and physiological variables** time-domain HRV indices (e.g., root mean square of successive differences [RMSSD], standard deviation of normal-to-normal intervals [SDNN], percentage of successive R–R intervals > 50 ms [pNN50]); frequency-domain HRV indices (e.g., LF, HF, LF/HF ratio); non-linear and geometric HRV indices (e.g., triangular index [TRI], triangular interpolation of NN interval histogram [TINN]); cardiovascular measures (e.g., blood pressure variability [BPV], systolic [SBP], diastolic [DBP], mean pressure [MP], pulse pressure [PP]); other autonomic markers (e.g., RSA, EDA/SCR, salivary cortisol, respiration rate

In addition to conventional autonomic markers, the review considered derived indices from physiological signals, including measures of physiological coupling and synchrony, such as time–frequency coherence (TFC), cross-frequency coupling (CFC), partial time–frequency coherence (pTFC), absolute coupling index (ACI), phase synchronization index (PSI), Integrative Coupling Index (ICI), or Granger Causality (GC); as well as geometric measures of HRV, including triangular index (TRI) and triangular interpolation of NN interval histogram (TINN).

- **Vocal task and context**: type of vocalization (e.g., sustained phonation, singing, reading aloud, polyphonic ensemble singing, speech under cognitive load), and whether the task was performed in an ecological or experimental setting.
- **Main findings**: outcomes related to changes in autonomic markers, voice parameters, or significant correlations presented by the original authors.
- **Key patterns**: (author-generated): concise statements synthesizing the main findings of each study on voice–ANS interactions, derived from the thematic analysis.

All information was extracted directly from the primary sources. Only objective data explicitly reported in the original studies were included, and the charted content was limited to descriptive elements such as population characteristics, vocal tasks, physiological variables, and outcomes relevant to the voice–ANS interaction. No critical appraisal of the included studies was conducted.

The extracted data were summarized using a structured descriptive approach. Each study was handled individually and presented in a uniform narrative format based on the predefined variables. This process facilitated the identification of recurring patterns through a descriptive synthesis, without quantitative comparison across studies.

## 3. Results

### 3.1. Identification and Selection of Studies

A total of 6097 records were identified through searches in five electronic databases: PubMed (n = 1953), Scopus (n = 2678), Web of Science (n = 1441), Embase (n = 22), and CINAHL (n = 3). After applying automatic filters for publication year, language, and population within each platform, 5703 records were excluded. The remaining 394 citations were screened by title and abstract, leading to the exclusion of 338 records that did not meet the eligibility criteria.

The remaining 56 citations were exported to Mendeley Reference Manager, where 23 duplicates were identified and removed. A total of 33 full-text articles were assessed for eligibility. Eighteen studies were excluded for the following reasons: no voice–ANS relationship (n = 11), ineligible population (n = 2), preliminary or non–peer-reviewed publication status (n = 2), or lack of active vocalization (n = 3).

**Figure 1:**
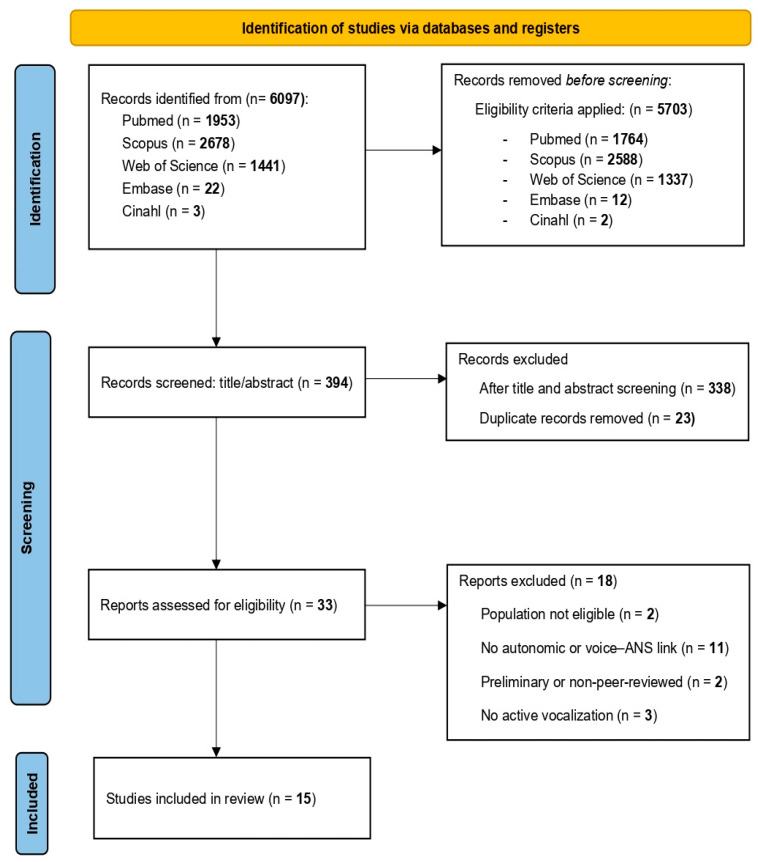
PRISMA 2020 flow diagram of study selection.

This resulted in a final sample of 15 included studies. Three of the full-text articles initially retained were later excluded during the data charting phase, as they did not provide analysable evidence linking vocal activity with autonomic function. The complete selection process is illustrated in the PRISMA 2020 flow diagram (Figure 1).

### 3.2. Characteristics and Results of Included Studies

This scoping review includes 15 studies published between 2011 and 2024, that examine the relationship between vocal activity and ANS modulation in adult populations. Most involved healthy individuals with high vocal demands—such as singers, actors, or performing arts students—while others assessed autonomic responses during vocal tasks under cognitive or emotional load.

Table 2 summarizes the main characteristics, autonomic variables, and reported outcomes of each included study. To complement these data, a set of key patterns for each study, providing concise statements that synthesize the main voice–ANS findings, which were subsequently organized into the thematic blocks of the discussion. Results are presented in chronological order and remain restricted to outcomes explicitly reported in the original publications that are relevant to the objectives of this review.

In a study with choir members, Müller and Lindenberger (2011) found synchronization of respiration and HRV during singing, most prominently during unison performances. The coupling occurred around 0.15 Hz (cycles per second, reflecting the boundary between the LF [0.04–0.15 Hz] and HF [0.15–0.40 Hz] bands of cardiorespiratory oscillations), and included a clear conductor-to-choir influence [24].

Sustained/æ/phonation during autonomic challenges increased HR more than BP, with F_0_ correlating specifically with HR. Bermúdez de Alvear et al. (2013) also observed that the strongest rises in both measures occurred during mental arithmetic [25].

Vocal tasks with different rhythmic structures elicited distinct autonomic responses. Vickhoff et al. (2013) demonstrated that mantra singing paced by a respiratory rhythm of 0.1 Hz produced the strongest increases in parasympathetic HRV indices, with pronounced HRV coherence, while hymn singing induced moderate effects and humming was not associated with interpersonal synchrony [26].

During oral exam stress, mean and minimum F_0_ increased in both spontaneous and read speech, and these changes were associated with elevated salivary cortisol concentrations, with no effect on maximum F_0_ or variability, as shown by Pisanski et al. (2016) [27].

Speech under cognitive stress was associated with increases in sympathetic indices and subtle alterations in voice quality. These changes were documented by MacPherson and colleagues (2017), who found that associations between autonomic and vocal measures did not reach statistical significance [28].

When comparing different vocalizations, Bernardi et al. (2017) documented that toning elicited stronger autonomic responses than song singing. Toning increased HRV, promoted a spontaneous breathing rhythm near 0.1 Hz, and HR rose similarly to singing [29].

Müller et al. (2019) identified negative correlations between HR, LF/HF ratio and CFC measures during canon singing. In the unison condition, both HR and LF/HF decreased with stronger CFC, whereas in the multipart condition, LF/HF decreased with increasing CFC input, indicating distinct autonomic patterns across singing modes [30].

In individuals with non-phonotraumatic vocal hyperfunction (NPVH), Ciccarelli et al. (2019) measured EDA and analysed SCR in relation to the standard deviation of fundamental frequency (F_0_ SD) in daily speech, finding significant associations with a 2 min delay [31].

In dyads of non-expert singers, long-note vocalizations increased inter-dyad HRV coherence. Partial TFC (pTFC) remained significant, even after accounting for RSA, and HR remained stable, with no significant changes, as reported by Ruiz-Blais et al. (2020) [32].

According to Tanzmeister et al. (2022), vocalization aligned with a 0.1 Hz respiratory cycle increased LF-HRV, HR, and SBP, with no change in HF-HRV [33].

In Lange et al. (2022), ensemble singing was associated with higher HRV synchrony, while physical contact enhanced respiratory phase synchronization without altering HRV [34].

SCR increased and Pulse Volume Amplitude (PVA) decreased in high-load speech tasks, as Abur et al. (2023) described, while acoustic parameters (CPP, L/H ratio, F_0_) showed no significant changes [35].

In adults without vocal complaints, Szkiełkowska et al. (2023) identified subclinical hyperfunctional dysphonia (subHD) in 26 cases, characterized by increased surface electromyography (sEMG) in the submental (SUB) and sternocleidomastoid (SCM) muscles, elevated HRV, reduced BVP, and higher EDA in singers [36].

During polyphonic ensemble singing, Scherbaum & Müller (2023) reported HRV synchronization in the top and middle voices, while the bass voice showed reduced variability and no synchronization [37].

In emotionally loaded phonation tasks, Krasnodębska et al. (2024) detected correlations between SUB and cricothyroid (CT) muscle activity and autonomic markers (HRV, EDA, BVP), whereas no associations were detected for sternocleidomastoid (SCM) [38].

## 4. Discussion

The studies included in this scoping review consistently demonstrate that vocalization engages the ANS across diverse contexts, including collective singing, structured and improvised vocal tasks, cognitively demanding speech, and clinical or subclinical dysphonia. Changes in HRV, EDA, BVP and related parameters were repeatedly documented, with task type and participant profile shaping the magnitude and direction of responses. A recurrent theme is the occurrence of physiological synchronization during collective vocalization. While this concept has sometimes been loosely applied to parallel physiological changes, in the present review it refers more specifically to the temporal alignment and coupling of autonomic rhythms (e.g., HRV and respiration) across individuals, exceeding what would be expected from coincident task-related arousal alone. Additionally, studies provided evidence that rhythm, temporal regularity, and vocal continuity modulate respiratory–cardiac coupling and autonomic markers (e.g., HRV, RSA), highlighting the regulatory potential of slow and repetitive vocalization. Evidence from stress paradigms points to bidirectional coupling between autonomic arousal and vocal output, while clinical and subclinical studies link laryngeal muscle activity with autonomic imbalance. Considered together, these findings support the active involvement of the ANS in vocal behavior and provide a framework for the thematic discussion that follows.

### 4.1. Autonomic Synchronization During Group Vocalization

Study 1 (Table 2) evidenced that group singing promotes interpersonal autonomic synchronization, with stronger respiratory and HRV coupling during unison (all singers on the same line) than in polyphony (independent vocal lines) or canon (sequential imitation). These findings underline that musical structure shapes physiological alignment and also revealed a social dimension, as the conductor exerted influence and singers clustered into synchrony modules by vocal part. Thus, synchronization extends beyond shared breathing and incorporates hierarchical and structural features of ensemble performance. This interpretation is consistent with the neurovisceral integration model, which proposes that HRV reflects the flexibility of central–peripheral networks involved in coordination [8]. Building on this evidence, Study 7 (Table 2) demonstrated that choral arrangement also determines the autonomic profile of synchronization. While unison was associated with stronger global synchrony and reductions in HR and LF/HF (indicating parasympathetic dominance), canon singing involved denser cardiorespiratory interactions and further LF/HF decreases, suggesting more complex sympathetic–parasympathetic distributions. Singers performing the same canon part were also found to have more efficient physiological networks than randomly assembled groups, indicating that musical structure can be directly mirrored in autonomic coordination patterns. Resonating with the autonomic imbalance framework, these results highlight parasympathetic dominance and HRV increases as markers of efficient regulatory capacity [39]. In addition to these experimental findings, Study 14 (Table 2) examined traditional Georgian polyphony in an ecological context and revealed that HRV synchronization was unevenly distributed across the ensemble. Upper and middle voices, characterized by greater expressive variation, displayed interpersonal coupling, whereas the bass line, more stable and supportive, showed little variability and no synchronization. These results indicate that, beyond structural arrangements such as unison or canon, the expressive and functional role of each vocal line also determines the distribution of autonomic alignment within the ensemble. This aligns with evidence that vagal modulation is anatomically differentiated across functional contexts, supporting role-specific autonomic responses [40]. However, all three studies relied on small, homogeneous samples and focused mainly on HR and HRV, without complementary autonomic markers or controlling for potential confounders. These limitations reduce robustness and highlight the need for larger, multimodal investigations.

Beyond structural arrangements in large ensembles, dyadic singing tasks provide further insight into autonomic synchronization. Study 9 (Table 2) revealed that sustained vocalization in non-expert singers induced significant increases in HRV coherence, largely mediated by shared respiration, but persisting after controlling for respiratory influences with pTFC. This suggests that joint phonation engages additional pathways of autonomic alignment beyond respiratory entrainment, consistent with evidence that RSA modulation during social interaction reflects regulatory processes that cannot be fully explained by respiration alone [14]. HR remained stable across conditions, indicating a fine-tuned modulation of autonomic variability rather than generalized cardiovascular arousal. Notably, peaks of HRV coherence coincided with vocal onsets, including frequencies exceeding conventional HRV bands (>0.4 Hz), underscoring that analyses restricted to standard ranges may underestimate key aspects of interpersonal dynamics. Extending these findings, Study 11 (Table 2) examined a professional ensemble performing polyphonic repertoire and confirmed that synchronization also occurs in musically demanding contexts. Its novel contribution was the introduction of direct physical contact (placing a hand on a neighbour’s shoulder or waist) to test the role of somatosensory cues. Contact significantly enhanced respiratory phase synchronization, but did not affect HRV coherence. This dissociation suggests selective responses: respiration, closely linked to motor and timing cues, is highly sensitive to tactile input that amplifies group awareness, whereas HRV coherence depends primarily on coordinated vocal production and remains stable despite external signals. The effect of touch was most pronounced when the full ensemble sang simultaneously, reinforcing that physiological alignment arises from the integration of vocal, motor, and social signals under conditions of maximal collective engagement. Similar dissociations can occur in social contexts, where RSA does not always parallel emotional expression [41].

Within this block, the studies converge on the relationship between collective vocalization and autonomic synchronization. The following key patterns reflect the specific contributions of each investigation:

- Unison singing elicits stronger autonomic synchronization than polyphony or canon.
- Canon singing amplifies cardiorespiratory coupling, unlike the parasympathetic dominance of unison.
- Vocal roles in polyphony determine how autonomic alignment is distributed across the ensemble.
- Sustained vocalization in dyads strengthens HRV coherence beyond respiratory influences.
- Physical contact during ensemble singing enhances respiratory synchronization while HRV coherence remains stable.

### 4.2. Autonomic Regulation Across Structured, Improvised, and Paced Vocalizations

The temporal organization of vocalization acts as a biological determinant of HRV and RSA. Study 3 (Table 2) showed that structured tasks, such as mantra chanting, paced repetition of a syllable at a 0.1 Hz respiratory rhythm, produced the strongest and most persistent synchronization, whereas humming, continuous vocalization on a single pitch, and hymn singing, structured melodic vocalization with lyrics, elicited weaker or more variable effects. Complementing these results, Study 6 (Table 2) demonstrated that toning—defined as the prolonged improvisation of vowel sounds without melodic or rhythmic structure—spontaneously slowed respiration towards 0.1 Hz, increased SDNN and LF-HRV, and promoted a cardiorespiratory profile comparable to formal slow-breathing techniques. Taken together, these findings indicate that both structured and unstructured vocalization can entrain autonomic regulation through respiratory pathways, favoring parasympathetic dominance Via RSA-mediated mechanisms, although methodological limitations such as the use of portable devices with limited temporal resolution should be acknowledged [42,43]. In contrast, Study 10 (Table 2) documented that paced singing at a respiratory rhythm of 0.1 Hz elicited a dual autonomic response, combining parasympathetic modulation with sympathetic activation, reflected in increased LF-HRV, HR, and systolic blood pressure. This finding is particularly relevant because slow breathing at 0.1 Hz is generally considered an optimal resonance pattern for baroreflex sensitivity and parasympathetic regulation [44]. The requirement for explicit pacing likely imposed additional cognitive and motor demands, since vocal sound production involves active muscular contractions that can recruit sympathetic pathways [45]. These findings highlight that, although paced singing can facilitate parasympathetic entrainment and enhance positive affect, its strict implementation may also increase cardiovascular load, indicating the need for cautious application in practical contexts.

Evidence indicates that variations in vocalization tasks modulate autonomic activity, engaging respiratory–cardiac mechanisms in specific ways. These dynamics are captured in the following key patterns, which reflect the contributions of each study:

- Mantra chanting coordinated with a 0.1 Hz respiratory rhythm maximizes HRV and RSA synchronization.
- Toning drives breathing towards ~0.1 Hz and increases HRV (SDNN, LF-HVR power).
- Paced singing at 0.1 Hz raises LF-HRV, but also elevates HR and BP, showing dual autonomic activation.

### 4.3. Cognitive and Emotional Stressors in Voice: Autonomic and Phonatory Responses

Cognitive and emotional demands during vocalization represent a critical interface where autonomic activation and laryngeal control converge. Study 2 (Table 2) demonstrated that sustained phonation during classical autonomic challenges was accompanied by increases in both HR and BP, with a significant association between F_0_ and HR observed at rest as well as under stress conditions. Among the stressors applied, mental arithmetic emerged as the most demanding, producing the largest joint increases in HR and F_0_. This finding indicates that cognitive load elicits a more complex autonomic activation than physical stressors, engaging cortical networks together with sympathetic–adrenergic mechanisms. The convergence of cardiac dynamics and laryngeal control suggests that fundamental frequency not only reflects phonatory activity, but also conveys, in real time, the imprint of autonomic state. Consistent with this view, it has been proposed that cardiac activity exerts a recurrent influence on vocal fold vibration, providing a physiological pathway for such coupling [17]. Nevertheless, Study 2 was limited by its small sample size and reliance on brief, laboratory-based phonation tasks without endocrine measures. Study 4 (Table 2) extended this evidence to a more ecological context of oral exam stress, demonstrating that increases in mean and minimum F_0_ were specifically associated with elevated cortisol concentrations. Notably, these F_0_ increases were evident primarily in participants whose cortisol responses exceeded twice their baseline values, underscoring the role of the hypothalamic–pituitary–adrenal (HPA) axis in vocal modulation. Thus, voice does not uniformly reflect stress, but rather appears sensitive to the magnitude of the endocrine response. This pattern is consistent with the view that cognitive–evaluative stressors trigger a coordinated activation of the HPA axis and the ANS and with evidence that individual differences in autonomic flexibility shape variability in stress reactivity [46,47].

Extending the line of evidence, Study 5 (Table 2) indicated that speech under cognitive stress was accompanied by increased sympathetic activation and subtle modifications in voice quality. Higher SCR were paired with changes in spectral–cepstral indices of phonation, indicating that autonomic activation can influence fine-grained aspects of vocal output, even when F_0_ and intensity remain stable. Evidence from cognitive conflict paradigms confirms that Stroop tasks reliably elicit sympathetic activation, while findings from ecological performance settings demonstrate that evaluative stress amplifies sympathetic drive and reduces vagal tone [48,49]. These complementary studies reinforce the interpretation of Study 5, supporting the view that sympathetic activation plays a key role in shaping phonatory control during cognitively demanding speech. However, Study 12 (Table 2)**,** conducted in older women, revealed a different profile: increased skin conductance responses and reduced pulse volume amplitude—an indicator of peripheral vasoconstriction linked to sympathetic activation—signalled clear autonomic reactivity under cognitive load, yet no significant acoustic changes were observed. This finding suggests that ageing may reduce the sensitivity of vocal parameters to autonomic modulation, likely due to structural and functional transformations in the laryngeal and respiratory systems, as well as age-related acoustic changes documented in the literature [50]. Even so, the small sample size and the absence of direct physiological measures of the phonatory system constrain interpretation.

Across the investigations included in this section, cognitive demands emerge as a central factor shaping how autonomic activity interacts with vocal control. The following key patterns outline the distinctive contributions of each investigation to this evidence:

- Mental arithmetic during phonation elicits strong HR–F_0_ coupling, highlighting cognitive load in autonomic–phonatory interaction.
- Exam stress raises F_0_ with elevated cortisol, linking HPA reactivity to autonomic–phonatory coupling.
- Cognitive stress during speech induces sympathetic activation and subtle voice quality changes beyond F_0_.
- In older women, cognitive load produces sympathetic activation without vocal change, revealing age-related voice–ANS decoupling.

### 4.4. Autonomic Signatures of Vocal Effort and Dysphonia

In individuals with non-phonotraumatic vocal hyperfunction (NPVH), Study 8 (Table 2) revealed a significant coupling between sympathetic activity (SCR) and F_0_ variability during daily speech, with a temporal lag of approximately two minutes. This delayed association suggests that autonomic activation is not immediately reflected in the voice, but rather modulates phonatory variability in a deferred manner. Although correlation values were modest, the finding suggests that in vocal hyperfunction, voice–ANS interaction may manifest subtly and with delay, supporting the view that psychophysiological stress perpetuates the hyperfunctional pattern. Additional support comes from self-report studies indicating that individuals with functional dysphonia report a higher prevalence of autonomic symptoms compared with controls [23,51].

Extending this line of evidence, Study 13 (Table 2) identified subclinical hyperfunctional dysphonia (subHD) in adults without vocal complaints, with increased extralaryngeal activation (SUB and SCM) and autonomic alterations—higher HRV, reduced BVP, and elevated EDA in singers. Although perceptual voice quality was normal, the correlations between muscle tension and autonomic variability suggest that laryngeal hyperfunction may be modulated by the ANS, even before clinical symptoms emerge. From a physiological perspective, these patterns may reflect an autonomic imbalance with sympathetic predominance, promoting extralaryngeal hypertonia and less efficient phonorespiratory coordination, in line with evidence that the central nervous system regulates laryngeal musculature through intertwined autonomic and motor circuits, explaining how sympathetic dominance can influence phonatory patterns [52]. In the same direction, Study 15 (Table 2) reported consistent correlations between laryngeal muscle activity (CT and SUB) and autonomic markers (HRV, EDA, BVP) during free phonation and glissando tasks, while SCM involvement was limited to higher phonatory demands, underscoring its role as an accessory respiratory muscle. The clearer correlations observed in professional singers indicate that vocal training, while acoustically compensatory, does not prevent the recruitment of accessory muscles under strain, a phenomenon also documented in healthy trained singers [53]. Taken together, these findings raise the possibility that vocal training may mask or delay the clinical manifestation of functional dysphonia.

These converging findings can be summarised as key patterns that link vocal effort with autonomic signatures:

- In NPVH, daily speech presents delayed SCR–F_0_ coupling, evidencing deferred voice–ANS interaction.
- SubHD exhibits extralaryngeal tension with autonomic imbalance, indicating early voice–ANS coupling.
- Laryngeal muscle activity (CT, SUB) couples with autonomic markers, evidencing voice–ANS interaction.

## 5. Limitations

Although this scoping review was designed with a rigorous methodological framework, certain limitations should be acknowledged. In line with PRISMA-ScR guidelines, no formal critical appraisal of the included studies was conducted, as the primary aim was to map existing evidence rather than assess study quality. Nonetheless, the inclusion of studies with heterogeneous designs and small sample sizes may influence the consistency of the associations identified. Additionally, gray literature and unpublished sources were excluded, which may have limited the retrieval of other relevant findings. The wide variability in vocal tasks, autonomic parameters, and methodological approaches across studies posed challenges for direct comparisons; however, this heterogeneity was mitigated through thematic synthesis in the discussion.

A further limitation is the intrinsic variability of some autonomic indices: for example, EDA and peripheral temperature are sensitive to skin and vascular factors, while HRV is influenced by demographic and clinical characteristics. These aspects should be considered when interpreting results, and future studies should apply appropriate methodological controls. A notable limitation is that most of the included studies focused on singing rather than speaking. Since these tasks differ in important physiological and functional ways, further research should specifically address speaking voice to provide a more comprehensive understanding of autonomic–vocal interactions.

Another physiological factor concerns respiration, which is fundamental to both vocal production and autonomic regulation and was inconsistently assessed across studies. It was systematically recorded in synchronization and paced vocalization tasks, but was largely absent from stress-related and dysphonia-focused investigations, restricting the ability to determine whether observed voice–ANS associations reflect genuine coupling or are partly mediated by respiratory influences. Finally, the interpretation of physiological synchronization is further challenged by inconsistent terminology across studies, which complicates comparison and underscores the importance of developing clearer conceptual and methodological standards to advance research on voice–ANS interactions.

## 6. Conclusions and Future Directions

This scoping review systematically mapped the current evidence on the relationship between voice production and ANS interactions in adult populations. Across diverse contexts, including group singing, structured and improvised tasks, cognitively demanding speech, and clinical or subclinical dysphonia, vocalization was consistently accompanied by measurable autonomic responses (HRV, EDA, BVP, HR, BP). The magnitude and direction of these responses varied according to task structure, emotional or cognitive load, and participants’ vocal profiles.

Considered together, these findings support the active involvement of the ANS in vocal behavior and provide a framework for the thematic discussion that follows. A distinctive feature of this review is that only studies measuring vocal and autonomic parameters simultaneously were considered, providing a synthesis grounded in direct evidence of voice–ANS coupling. Four thematic trends emerged from the literature: (1) group vocalization can induce interpersonal autonomic synchronization, strongly mediated by respiration and task structure, (2) slow-paced and rhythmically structured organized vocalizations modulate parasympathetic activity via respiratory coupling, while paced singing may impose additional sympathetic load, (3) emotional and cognitive vocal challenges reveal context-dependent interactions between vocal output, autonomic activity, and endocrine responses, and (4) hyperfunctional and subclinical dysphonia profiles are consistently associated with autonomic imbalance, suggesting early markers of dysfunction.

Despite methodological heterogeneity, the evidence converges on the notion that the ANS is not merely reactive, but actively contributes to the regulation, adaptation, and potential dysregulation of vocal behavior. These insights highlight the value of incorporating autonomic measures into both experimental voice research and clinical voice evaluation, particularly in functional or effort-related dysphonia.

Future studies should prioritize methodological standardization, systematically include respiration measures, and broaden the scope beyond singing to encompass spoken voice. Expanding research into clinical populations with functional dysphonia and high vocal load will be crucial for validating autonomic markers as diagnostic or prognostic tools. Comparative designs using structured vocal tasks may help delineate physiological signatures linked to distinct vocal profiles. Ultimately, integrating objective autonomic assessment into voice evaluation holds promise for earlier detection of dysfunction, refinement of diagnoses, and development of personalized clinical and occupational interventions.

## Figures and Tables

**Table 1:** Keywords used in the search strategy, organized by PCC component.

PCC	Keywords
(P)	“Professional Voice Users”, “Teachers”, “Singers”, “Voice Disorders”, “Functional Dysphonia”, “Muscle Tension Dysphonia”, “Vocal Hyperfunction”
(C)	“Voice”, “Singing”, “Voice production”, “Vocal effort”, “Autonomic Nervous System”, “Autonomic Dysfunction”, “Autonomic Regulation”, “Sympathetic Nervous System”, “Parasympathetic Nervous System”, “Heart Rate Variability”, “HRV”, “Heart Rate”, “Heartbeat”, “Blood Pressure”, “Stress Response”
(C)	“Structured vocal tasks”, “Singing Ensemble”, “Singing performance”, “Spoken tasks under stress”, “Cognitive-emotional vocal conditions”, “Experimental voice protocols”

**Table 2:** Overview of the studies included in the scoping review on the relationship between voice production and autonomic nervous system function.

No.	First Author (Year)	Study Design	Study Population	Study Objective	Autonomic Variables Assessed	Vocal Task	Main Findings	Key Patterns
**1** [24]	Müller & Lindenberger (2011)	Controlled observational within-subject	n = 12 (11 singers + 1 conductor); adult choir members (Germany)	To examine how choral singing influences interpersonal synchronization of autonomic and respiratory signals, comparing unison with multipart vocal conditions.	HRV synchrony (PSI, ACI, ICI, GC), respiration (PSI, ACI, ICI, GC)	Unison singing, part singing, and canon with eyes open and closed	Respiration and HRV synchronization increased during singing (>0.15 Hz), strongest in unison (η^2^ = 0.83; 0.59); GC showed conductor influence (*p* < 0.0001).	**Unison singing elicits stronger autonomic synchronization than polyphony or canon.**
**2** [25]	Bermúdez de Alvear et al. (2013)	Controlled experimental	n = 14 healthy adults (7 men, 7 women) without known vocal or cardiovascular conditions (Spain)	To determine whether voice F_0_ correlates with HR and blood pressure during autonomic challenge.	HR, SP, DP, MP	Sustained/æ/phonation (5 s) during baseline and three autonomic tasks: handgrip, cold pressor and arithmetic.	Phonation increased HR and MBP across conditions, with ΔHR exceeding MBP changes. F_0_ correlated with HR during phonation (r = 0.290; *p* < 0.001), but not with BP. Mental arithmetic triggered the highest HR and F_0_ rise (~13 Hz).	**Mental arithmetic during phonation elicits strong HR–F_0_ coupling, highlighting cognitive load in autonomic–phonatory interaction.**
**3** [26]	Vickhoff et al. (2013)	Controlled experimental	n = 11; healthy 18-year-olds with choral experience (Sweden)	To characterize the autonomic response to vocal tasks with varying respiratory and rhythmic structures.	HRV (RMSSD, coherence), RSA, HR, SC, temperature	Humming, hymn singing, mantra singing (respiratory rhythm 0.1 Hz)	Mantra: ↑RMSSD (*p* < 0.01), highest HRV coherence; Hymn: ↑RMSSD (*p* < 0.05), moderate coherence; Humming: no group synchrony; SC and temp: no change.	**Mantra chanting coordinated with a 0.1 Hz respiratory rhythm maximises HRV and RSA synchronization.**
**4** [27]	Pisanski et al. (2016)	Controlled experimental	n = 34; female undergraduate psychology students (United Kingdom)	To assess whether individual cortisol reactivity predicts changes in voice pitch during academic oral exam stress.	Salivary cortisol	Spontaneous and read speech (oral exam context)	↑Mean and min F_0_ under stress in both tasks (*p* = 0.014/0.034); cortisol ↑ (+74%) predicted F_0_ only under stress (rs = 0.46/0.45); no effect on -max F_0_ or SD.	**Exam stress raises F_0_ with elevated cortisol, linking HPA reactivity to autonomic–phonatory coupling.**
**5** [28]	MacPherson et al. (2017)	Controlled experimental within-subject	n = 16; healthy young adults (USA)	To analyse the effects of cognitive load during speech on autonomic arousal and vocal acoustics.	SCR, PVA, PP	Oral reading of Stroop stimuli (congruent vs. incongruent conditions)	Cognitive load during speech increased sympathetic arousal (↑SCR, *p* = 0.001) and altered voice quality (↑CPP, *p* = 0.050; ↓L/H ratio, *p* = 0.004)	**Cognitive stress during speech induces sympathetic activation and subtle voice quality changes beyond F_0_.**
**6** [29]	Bernardi et al. (2017)	Controlled crossover experimental	n = 20 healthy adults; no vocal training (Canada)	To examine the cardiorespiratory effects of song singing and toning and clarify whether observed changes stem from vocalization itself or the associated breathing pattern.	HRV (SDNN, LF, HF), HR	Singing of familiar slow songs (Western style) and improvised vocalization of free vowel sounds (toning)	Toning increased HRV (SDNN: *p* < 0.001, $\eta_{p}^{2}$ = 0.70) and LF power (*p* < 0.001, $\eta_{p}^{2}$ = 0.48), while reducing HF power (*p* < 0.001, $\eta_{p}^{2}$ = 0.57), compared to singing. HR rose in both tasks (*p* = 0.002, $\eta_{p}^{2}$ = 0.41).	**Toning drives breathing towards ~0.1 Hz and increases HRV (SDNN, LF-HRV power).**
**7** [30]	Müller et al. (2019)	Controlled within-subject experimental	n = 12; adults amateur choir members (Germany)	To characterize the changes in network topology induced by choral singing and their association with HR and HRV as measures of autonomic activity.	HR, HRV (SDNN, RMSSD, LF/HF)	Canon singing in unison (*Cun*); canon singing in three parts with eyes open (*Ceo*)	In *Cun*, HR and LF/HF decreased with stronger CFC input and output (r = −0.799; r = −0.667). In *Ceo*, LF/HF decreased with CFC input (r = −0.576)	**Canon singing amplifies cardiorespiratory coupling, unlike the parasympathetic dominance of unison.**
**8** [31]	Ciccarelli et al. (2019)	Longitudinal observational (ambulatory)	n = 14 adults with NPVH (USA)	To characterize SCR–F_0_ SD coupling in patients with NPVH during daily voice use.	EDA (SCR)	Ambulatory speech in daily life	Significant SCR—F_0_ SD correlations (*p* < 0.05) were predominantly detected at a 2 min lag in NPVH group.	**In NPVH, daily speech presents delayed SCR–F_0_ coupling, evidencing deferred voice–ANS interaction.**
**9** [32]	Ruiz-Blais et al. (2020)	Controlled experimental (within-subjects)	n = 18; non-expert singers (United Kingdom)	To determine whether vocal tasks induce HRV synchrony in non-experts, and if this coupling exceeds the effects of respiration.	HR, RMSSD; HRV inter-dyad coherence (TFC, pTFC)	Synchronized short, synchronized long, and asynchronous short notes.	↑HRV TFC and RMSSD during long-note vocalizations (*p* = 0.0039 and *p* = 0.0002); ↑pTFC (*p* = 0.0078) after controlling for RSA; no HR change.	**Sustained vocalization in dyads strengthens HRV coherence beyond respiratory influences.**
**10** [33]	Tanzmeister et al. (2022)	Randomized controlled experimental	n = 101; healthy amateur singers aged 18–44 (Austria)	To evaluate if paced singing at 0.1 Hz enhances cardiovascular regulation and reduces stress reactivity.	HR, LF-/HF-HRV, SBP, DBP	Paced singing at 0.1 Hz vs. Spontaneous singing	Paced singing (0.1 Hz): ↑LF-HRV (*p* < 0.001, *d* = 1.66); ↑HR (*p* < 0.001, *d* = 1.23); ↑SBP (*p* < 0.001, *d* = 1.48); no change in HF-HRV.	**Paced singing at 0.1 Hz raises LF-HRV, but also elevates HR and BP, showing dual autonomic activation.**
**11** [34]	Lange et al. (2022)	Experimental within-subject	n = 9; healthy adult professional singers and a male conductor (Germany)	To test the impact of physical contact on cardiorespiratory synchronization during ensemble singing.	HRV (PSI, ACI, ICI), respiration (PSI, ACI, ICI)	Ensemble singing with and without physical contact	Singing ↑HRV synchronization (PSI η^2^ = 0.568, *p* = 0.019); Touch vs. no touch: ↑respiration synchronization with touch (PSI η^2^ = 0.539, *p* = 0.024); no touch effect on HRV.	**Physical contact during ensemble singing enhances respiratory synchronization while HRV coherence remains stable.**
**12** [35]	Abur et al. (2023)	Prospective observational	n = 12 (6 males, 6 females) healthy older adults (68–78 years of age) (USA)	To assess the impact of cognitive load on autonomic activation and voice acoustics during structured speech tasks.	PVA, PP, SCR	Reading Stroop sentences aloud (congruent vs. incongruent conditions)	During vocal tasks under cognitive load, SCR amplitude increased (*p* < 0.001) and pulse volume amplitude decreased (*p* = 0.025). No significant changes were observed in acoustic measures (CPP, L/H ratio, F_0_).	**In older women, cognitive load produces sympathetic activation without vocal change, revealing age-related voice–ANS decoupling.**
**13** [36]	Szkiełkowska et al. (2023)	Cross-sectional observational	n = 81; 27 operas singers and 54 controls; healthy, no voice complaints (Poland)	To assess whether SEMG and ANS parameters can detect early signs of hyperfunctional dysphonia.	HRV, BVP, EDA	Sustained/æ/ phonation and glissando	↑SEMG amplitude in subHD (SUB, max = 254 mV, and SCM, max = 201 mV); ↑HRV, ↓BVP, ↑EDA (only in singers)	**SubHD exhibits extralaryngeal tension with autonomic imbalance, indicating early voice–ANS coupling.**
**14** [37]	Scherbaum & Müller (2023)	Observational study (with experimental component)	n = 3 professional male singers (Georgia)	To investigate HRV synchronization during polyphonic singing.	HRV (RMSSD)	Polyphonic ensemble singing (Georgian tradition)	Two singers (top and middle voices) exhibited synchronized HRV patterns during singing; whereas the bass voice displayed less variability and no clear synchrony.	**Vocal roles in polyphony determine how autonomic alignment is distributed across the ensemble.**
**15** [38]	Kranodębska et al. (2024)	Cross-sectional observational	n = 50 adults: 26 operas singers and 24 controls; all vocally healthy (Poland)	To explore the association between vocal muscle activity and autonomic responses during vocal tasks	HRV (RMSSD, SDNN, SDSD, pNN50, TRI, TINN), HR, EDA, BVP	Free phonation and glissando	Free phonation and glissando, performed under emotional load, in the full sample, were associated with significant correlations (*p* < 0.05) between SUB and CT amplitudes and HRV (SDNN, RMSSD, pNN50, TRI), EDA and BVP. No association found for SCM	**Laryngeal muscle activity (CT, SUB) couples with autonomic markers, evidencing voice–ANS interaction.**

**Note**. Abbreviations and variable labels are reported as in the original studies and are listed approximately in order of appearance within the table: ***HRV*** = heart rate variability; ***PSI*** = phase synchronization index; ***ACI*** = absolute coupling index; ***ICI*** = integrative coupling index; ***GC*** = Granger causality; ***HR*** = heart rate; **SP/*SBP*** = systolic blood pressure; **DP/*DBP*** = diastolic blood pressure; ***MBP*** = mean pressure; ***F*_0_** = fundamental frequency; ***RMSSD*** = root mean square of successive differences; ***RSA*** = respiratory sinus arrhythmia; ***SC*** = skin conductance; ***f*_0_*SD*** = standard deviation of fundamental frequency; ***SCR*** = skin conductance response; ***PVA*** = pulse volume amplitude; ***PP*** = pulse pressure; ***L/H ratio*** = the low/high spectral ratio; ***SDNN*** = standard deviation of NN intervals; ***LF*** = low-frequency power; ***HF*** = high-frequency power; ***CFC*** = cross-frequency coupling; ***EDA*** = electrodermal activity; ***NPVH*** = non-phonotraumatic vocal hyperfunction; ***TFC*** = time–frequency coherence; ***pTFC*** = partial time–frequency coherence; ***CPP*** = cepstral peak prominence; ***BVP*** = blood volume pulse; ***SEMG*** = surface electromyography; ***subHD*** = subclinical hyperfunctional dysphonia; ***CT*** = cricothyroid; ***SUB*** = submental; ***SCM*** = sternocleidomastoid; ***SDSD*** = standard deviation of successive differences; ***pNN50*** = percentage of adjacent NN intervals differing by more than 50 ms; ***TRI*** = triangular index; ***TINN*** = triangular interpolation of NN interval histogram; ***n*** = number of participants; ***p*** = level of statistical significance (typically *p* ≤ 0.05); **r** = correlation coefficient (strength and direction of association between variables); **Δ** = difference or change; **η^2^** = eta squared (effect size); $\eta_{p}^{2}$ = partial eta squared (effect size); **↑** = increase; **↓** = decrease.

## Data Availability

No new data were created or analyzed in this study. Data sharing is not applicable to this article.

## Abbreviations

HRV	Heart Rate Variability
PSI	Phase Synchronization Index
ACI	Absolute Coupling Index
ICI	Integrative Coupling Index
GC	Granger Causality
HR	Heart Rate
BP	Blood Pressure
MP	Mean Pressure
F_0_	Fundamental Frequency
RMSSD	Root Mean Square of Successive Differences
RSA	Respiratory Sinus Arrhythmia
SC	Skin Conductance
F_0_ SD	Standard Deviation of Fundamental Frequency
SCR	Skin Conductance Response
PVA	Pulse Volume Amplitude
PP	Pulse Pressure
L/H ratio	Low/High Spectral Ratio
SDNN	Standard Deviation of NN Intervals
LF	Low-Frequency Power
HF	High-Frequency Power
CFC	Cross-Frequency Coupling
EDA	Electrodermal Activity
NPVH	Non-Phonotraumatic Vocal Hyperfunction
TFC	Time–Frequency Coherence
pTFC	Partial Time–Frequency Coherence
SBP	Systolic Blood Pressure
DBP	Diastolic Blood Pressure
CPP	Cepstral Peak Prominence
BVP	Blood Volume Pulse
SEMG	Surface Electromyography
subHD	Subclinical Hyperfunctional Dysphonia
CT	Cricothyroid
SUB	Submental
SCM	Sternocleidomastoid
SDSD	Standard Deviation of Successive Differences
pNN50	Percentage of Adjacent NN Intervals Differing by More Than 50 ms
TRI	Triangular Index
TINN	Triangular Interpolation of NN Interval Histogram

## References

[1] Chhetri D.K., Neubauer J., Sofer E., Berry D.A. (2014). Influence and interactions of laryngeal adductors and cricothyroid muscles on fundamental frequency and glottal posture control. J. Acoust. Soc. Am..

[2] Lã F.M.B., Gill B.P., Welch G., Howard D.M., Nix J. (2015). Physiology and its impact on the performance of singing. The Oxford Handbook of Singing.

[3] Benarroch E.E. (1993). The central autonomic network: Functional organization, dysfunction, and perspective. Mayo Clin. Proc..

[4] Achey M.A., He M.Z., Akst L.M. (2016). Vocal hygiene habits and vocal handicap among conservatory students of classical singing. J. Voice.

[5] Castelblanco L., Habib M., Stein D.J., Quadros A., Cohen S.M., Noordzij J.P. (2014). Singing voice handicap and videostrobolaryngoscopy in healthy professional singers. J. Voice.

[6] D’haeseleer E., Claeys S., Meerschman I., Bettens K., Degeest S., Dijckmans C., Smet J., Luyten A., Van Lierde K. (2017). Vocal characteristics and laryngoscopic findings in future musical theater performers. J. Voice.

[7] El-Demerdash A.M., Hafez N.G., Tanyous H.N., Rezk K.M., Shadi M.S. (2024). Screening of voice and vocal tract changes in professional wind instrument players. Eur. Arch. Oto-Rhino-Laryngol..

[8] Thayer J.F., Lane R.D. (2000). A model of neurovisceral integration in emotion regulation and dysregulation. J. Affect. Disord..

[9] Wehrwein E.A., Orer H.S., Barman S.M. (2016). Overview of the anatomy, physiology, and pharmacology of the autonomic nervous system. Compr. Physiol..

[10] Gibbons C.H., Low P.A., Benarroch E.E. (2019). Basics of autonomic nervous system function. Handbook of Clinical Neurology.

[11] Benarroch E.E. (2020). Physiology and pathophysiology of the autonomic nervous system. Contin. Lifelong Learn. Neurol..

[12] Lara J.P., Dawid-Milner M.S., López M.V., Montes C., Spyer K.M., González-Barón S. (2002). Laryngeal effects of stimulation of rostral and ventral pons in the anaesthetized rat. Brain Res..

[13] González-García M., Carrillo-Franco L., Morales-Luque C., Ponce-Velasco M., Gago B., Dawid-Milner M.S., López-González M.V. (2024). Uncovering the neural control of laryngeal activity and subglottic pressure in anaesthetized rats: Insights from mesencephalic regions. Pflügers Arch. Eur. J. Physiol..

[14] Butler E.A., Wilhelm F.H., Gross J.J. (2006). Respiratory sinus arrhythmia, emotion, and emotion regulation during social interaction. Psychophysiology.

[15] Berntson G.G., Bigger J.T., Eckberg D.L., Grossman P., Kaufmann P.G., Malik M., Nagaraja H.N., Porges S.W., Saul J.P., Stone P.H. (1997). Heart rate variability: Origins, methods, and interpretive caveats. Psychophysiology.

[16] Dawson M.E., Schell A.M., Filion D.L., Cacioppo J.T., Tassinary L.G., Berntson G.G. (2007). The electrodermal system. Handbook of Psychophysiology.

[17] Orlikoff R.F., Baken R.J. (1989). The effect of the heartbeat on vocal fundamental frequency perturbation. J. Speech Lang. Hear. Res..

[18] Helou L.B., Jennings J.R., Rosen C.A., Wang W., Verdolini Abbott K. (2020). Intrinsic laryngeal muscle response to a public speech preparation stressor: Personality and autonomic predictors. J. Speech Lang. Hear. Res..

[19] Motamed Yeganeh N., McKee T., Werker J.F., Hermiston N., Boyd L.A., Cui A.-X. (2024). Opera trainees’ cognitive functioning is associated with physiological stress during performance. Music. Sci..

[20] Cardoso R., Meneses R.F., Lumini-Oliveira J., Pestana P. (2021). Associations between teachers’ autonomic dysfunction and voice complaints. J. Voice.

[21] Dahl K.L., Stepp C.E. (2023). Effects of cognitive stress on voice acoustics in individuals with hyperfunctional voice disorders. Am. J. Speech Lang. Pathol..

[22] Schneider B., Enne R., Cecon M., Diendorfer-Radner G., Wittels P., Bigenzahn W., Johannes B. (2006). Effects of vocal constitution and autonomic stress-related reactivity on vocal endurance in female student teachers. J. Voice.

[23] Park K., Behlau M. (2011). Sinais e sintomas da disfunção autônoma em indivíduos disfônicos. J. Soc. Bras. Fonoaudiol..

[24] Müller V., Lindenberger U. (2011). Cardiac and respiratory patterns synchronize between persons during choir singing. PLoS ONE.

[25] Bermúdez Alvear R.M., Barón-López F.J., Alguacil M.D., Dawid-Milner M.S. (2013). Interactions between voice fundamental frequency and cardiovascular parameters. Preliminary results and physiological mechanisms. Logop. Phoniatr. Vocol..

[26] Vickhoff B., Malmgren H., Åström R., Nyberg G., Ekström S.-R., Engwall M., Snygg J., Nilsson M., Jörnsten R. (2013). Music structure determines heart rate variability of singers. Front. Psychol..

[27] Pisanski K., Nowak J., Sorokowski P. (2016). Individual differences in cortisol stress response predict increases in voice pitch during exam stress. Physiol. Behav..

[28] MacPherson M.K., Abur D., Stepp C.E. (2017). Acoustic measures of voice and physiologic measures of autonomic arousal during speech as a function of cognitive load. J. Voice.

[29] Bernardi N.F., Snow S., Peretz I., Orozco Perez H.D., Sabet-Kassouf N., Lehmann A. (2017). Cardiorespiratory optimization during improvised singing and toning. Sci. Rep..

[30] Müller V., Delius J.A.M., Lindenberger U. (2019). Hyper-frequency network topology changes during choral singing. Front. Physiol..

[31] Ciccarelli G., Mehta D., Ortiz A., Van Stan J., Toles L., Marks K., Hillman R., Quatieri T. Correlating an ambulatory voice measure to electrodermal activity in patients with vocal hyperfunction. Proceedings of the 2019 IEEE 16th International Conference on Wearable and Implantable Body Sensor Networks (BSN).

[32] Ruiz-Blais S., Orini M., Chew E. (2020). Heart rate variability synchronizes when non-experts vocalize together. Front. Physiol..

[33] Tanzmeister S., Rominger C., Weber B., Tatschl J.M., Schwerdtfeger A.R. (2022). Singing at 0.1Hz as a resonance frequency intervention to reduce cardiovascular stress reactivity?. Front. Psychiatry.

[34] Lange E.B., Omigie D., Trenado C., Müller V., Wald-Fuhrmann M., Merrill J. (2022). Intouch: Cardiac and respiratory patterns synchronize during ensemble singing with physical contact. Front. Hum. Neurosci..

[35] Abur D., MacPherson M.K., Shembel A.C., Stepp C.E. (2023). Acoustic measures of voice and physiologic measures of autonomic arousal during speech as a function of cognitive load in older adults. J. Voice.

[36] Szkiełkowska A., Krasnodębska P., Mitas A., Bugdol M., Romaniszyn-Kania P., Pollak A. (2023). Electrophysiological predictors of hyperfunctional dysphonia. Acta Oto-Laryngol..

[37] Scherbaum F., Müller M. (2023). From intonation adjustments to synchronization of heart rate variability: Singer interaction in traditional Georgian vocal music. Musicologist.

[38] Krasnodębska P., Szkiełkowska A., Pollak A., Romaniszyn-Kania P., Bugdol M.N., Bugdol M.D., Mitas A. (2024). Analysis of the relationship between emotion intensity and electrophysiology parameters during a voice examination of opera singers. Int. J. Occup. Med. Environ. Health.

[39] Thayer J.F., Yamamoto S.S., Brosschot J.F. (2010). The relationship of autonomic imbalance, heart rate variability and cardiovascular disease risk factors. Int. J. Cardiol..

[40] Thayer J.F., Sternberg E.M. (2009). Neural concomitants of immunity—Focus on the vagus nerve. Neuroimage.

[41] Frazier T.W., Strauss M.E., Steinhauer S.R. (2004). Respiratory sinus arrhythmia as an index of emotional response in young adults. Psychophysiology.

[42] Grossman P., Kollai M. (1993). Respiratory sinus arrhythmia, cardiac vagal tone, and respiration: Within- and between-individual relations. Psychophysiology.

[43] Grossman P., Stemmler G., Meinhardt E. (1990). Paced respiratory sinus arrhythmia as an index of cardiac parasympathetic tone during varying behavioral tasks. Psychophysiology.

[44] Schwerdtfeger A.R., Schwarz G., Pfurtscheller K., Thayer J.F., Jarczok M.N., Pfurtscheller G. (2020). Heart rate variability (HRV): From brain death to resonance breathing at 6 breaths per minute. Clin. Neurophysiol..

[45] Chin M.S., Kales S.N. (2019). Understanding mind-body disciplines: A pilot study of paced breathing and dynamic muscle contraction on autonomic nervous system reactivity. Stress Health.

[46] Dickerson S.S., Kemeny M.E. (2004). Acute stressors and cortisol responses: A theoretical integration and synthesis of laboratory research. Psychol. Bull..

[47] Friedman B.H., Thayer J.F. (1998). Autonomic balance revisited: Panic anxiety and heart rate variability. J. Psychosom. Res..

[48] Kobayashi N., Yoshino A., Takahashi Y., Nomura S. (2007). Autonomic arousal in cognitive conflict resolution. Auton. Neurosci..

[49] Moreno-Gutiérrez J.Á., Rojas Leal C., López-González M.V., Chao-Écija A., Dawid-Milner M.S. (2023). Impact of music performance anxiety on cardiovascular blood pressure responses, autonomic tone and baroreceptor sensitivity to a western classical music piano-concert. Front. Neurosci..

[50] Torre P., Barlow J.A. (2009). Age-related changes in acoustic characteristics of adult speech. J. Commun. Disord..

[51] Demmink-Geertman L., Dejonckere P.H. (2002). Nonorganic habitual dysphonia and autonomic dysfunction. J. Voice.

[52] Ludlow C.L. (2005). Central nervous system control of the laryngeal muscles in humans. Respir. Physiol. Neurobiol..

[53] Watson A.H., Williams C., James B.V. (2012). Activity patterns in latissimus dorsi and sternocleidomastoid in classical singers. J. Voice.

